# Remembering Keisuke Fujita, M.D., Ph.D., President and Founder of Fujita Health University, and his contributions to medical science and education

**DOI:** 10.20407/fmj.2020-031

**Published:** 2021-03-20

**Authors:** Toshiharu Nagatsu

**Affiliations:** 1 Center for Research Promotion and Support, Fujita Health University, Toyoake, Aichi, Japan; 2 Adviser, Fujita Health University, School of Medicine, Toyoake, Aichi, Japan

**Keywords:** President Dr. Keisuke Fujita, Fujita Health University School of Medicine, Scientific contributions

## Abstract

Keisuke Fujita, M.D., Ph.D. (1925–1995), founded and was President of Fujita Gakuen (academy) in 1964, Fujita Health University in 1968, and Fujita Health University (FHU) School of Medicine in 1972. He also established the Institute for Comprehensive Medical Science (ICMS) at FHU in 1972, at the same time as the founding of FHU School of Medicine, to promote the institutional development of FHU School of Medicine by providing a strong foundation for science. I collaborated with Dr. Fujita from 1965. Returning from the study abroad at the NIH in the USA in 1965, I joined Professor Fujita’s Department of Biochemistry at the Aichi Gakuin University School of Dentistry, as an Associate Professor. Dr. Fujita’s major research interest was in the biochemistry of diseases, namely, cancer, neuropsychiatric diseases, and various intractable diseases, which he investigated by applying analytical chemistry and molecular/cellular biochemistry. He was also interested in the pharmacognosy of aloe plants and established “Syoyaku Kenkyu Juku (Center for Pharmacognosy),” and where he studied by himself and trained many FHU graduates. I herein present an overview of the research carried out by Dr. Fujita to share his legacy and praise his memoir and contributions to medical science and education for all faculty members, staffs and students of FHU. It is assumed that individuals at FHU have already made significant contributions to medical science. I hope that his vision of FHU producing a Nobel Prize laureate will be realized someday.

## 1. Introduction

**Figure 1:**
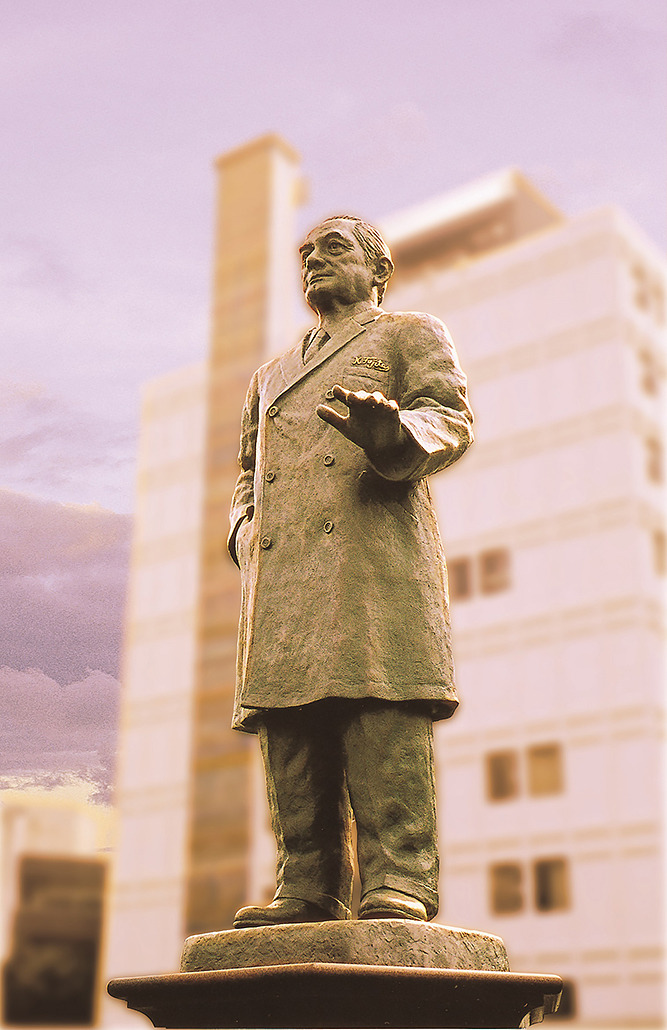
Bronze statue of President Dr. Keisuke Fujita, President of Fujita Gaken and Fujita Health University, on the School’s campus.

Keisuke Fiujita, M.D., Ph.D. (1925–1995), founded and was President of Fujita Gakuen (academy) in 1964, Fujita Health University College (FHU College) in 1966, Fujita Health University (FHU) in 1968, and FHU School of Medicine in 1972. Thus, during his life, Dr. Fujita established four schools, Fujita Gakuen (academy) in 1964, FHU College in 1966, FHU in 1968, and FHU School of Medicine in 1972 (Figure 1). He also established the Institute for Comprehensive Medical Science (ICMS) at FHU in 1972, at the same time as the founding of FHU School of Medicine by providing a strong foundation in science.

I collaborated with Dr. Fujita over 30 years from 1965. In 1965, shortly after returning to Japan from studying abroad, I took the position of the Associate Professor at the Department of Biochemistry, Aichi Gakuin University School of Dentistry, Nagoya City, where Dr. Fujita was the Professor and Department Chair.

**Figure 2:**
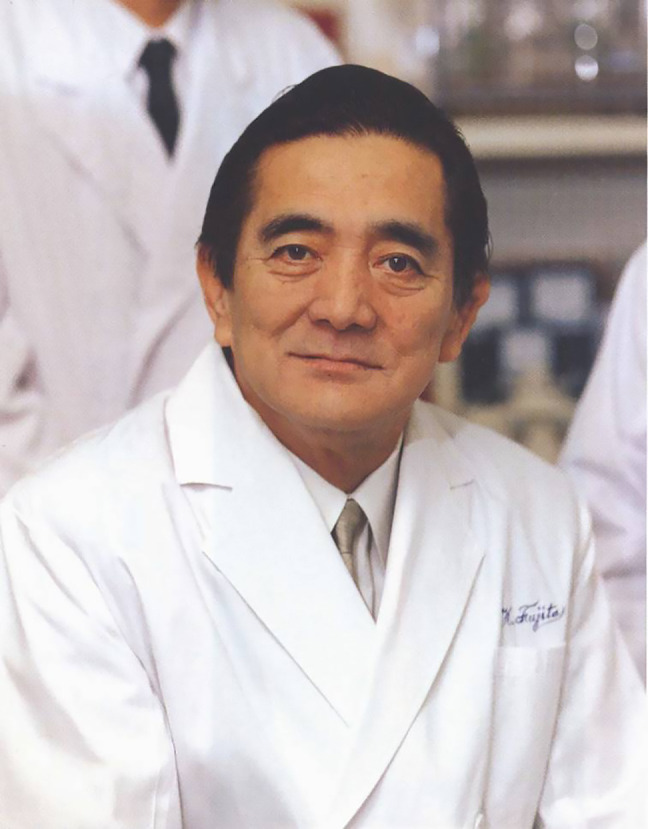
Dr. Keisuke Fujita in the laboratory.

Dr Fujita’s major research interest was in the biochemical study of diseases, namely, cancer, neuropsychiatric diseases, and various intractable diseases, which he investigated by applying analytical chemistry and molecular/cellular biochemistry. He was also interested in pharmacognosy of aloe plants and organized “Syoyaku Kenkyu Juku” (Center for Pharmacognosy), where he trained many graduates from FHU (Figure 2).

I herein present an overview in commemoration of Dr. Fujita’s life and his extraordinary contributions to medical science and education, to be shared with FHU students and faculty personnel, as well as the general public.

## 2. Personal history of Dr. Keisuke Fujita: Founding President of Fujita Gakuen (academy) and Fujita Health University

Dr. Keisuke Fujita was born in Okayama Prefecture in1925, and grew up in Niihama City, Ehime Prefecture, in Shikoku Island, Japan. He was graduated from the Nagoya University School of Medicine, Nagoya City, Aichi Prefecture, in 1948, and earned his M.D. degree. He earned his Ph.D. degree in the biochemistry from the Nagoya University Graduate School of Medicine in 1955. That same year, he became the Assistant Professor of Biochemistry at the Nagoya University School of Medicine. In 1960, he was invited to the Iwate Medical University, Morioka City, Iwate Prefecture, in the northeastern region of Japan, as the Associate Professor of Biochemistry, and as the adjunct Director of the Clinical Chemistry Laboratory, the first-of-its-kind in Japan. In 1963, he became the Full Professor of Biochemistry at Aichi Gakuin University School of Dentistry. In 1965, shortly after returning to Japan from studying abroad, I became the Associate Professor of Biochemistry at the Aichi Gakuin University School of Dentistry, where Dr. Fujita was the Professor of Biochemistry and Department Chair.

Dr. Fujita established Fujita Gakuen (academy) in Toyoake City in the eastern suburbs of Nagoya City, Aichi Prefecture in 1964. I clearly recall his words upon returning from a faculty meeting at Aichi Gakuin University School of Dentistry late at night on the last day of March 1966. He told me that he had decided to leave the Department of Biochemistry of the Aichi Gakuin University School of Dentistry, in order to start a new medical university of Fujita Gakuen in Toyoake City, and that he had recommended me to Aichi Gakuin University School of Dentistry as his successor as Professor of Biochemistry. He also said that we would be able to continue our research collaborations in the future. From that time, I had numerous research collaborations with Dr. Fujita on the projects of mutual interests up until his sudden death on June 11,1995. During these 30 years, I worked for the Aichi Gakuin University School of Dentistry, the Tokyo Institute of Technology Graduate School of Life Chemistry, the Nagoya University School of Medicine, and finally joined the Institute for Comprehensive Medical Science (ICMS), FHU in 1991. I also worked in the USA, for nearly 4 years at: National Institutes of Health (NIH) in Bethesda, Maryland, the University of Southern California School of Medicine in Los Angeles, California, and the Roche Institute of Molecular Biology in Nutley, New Jersey.

As described above, during his lifetime, Dr. Fujita established four schools, Fujita Gakuen (academy) in 1964, FHU College in 1966, FHU in 1968, and FHU School of Medicine in 1972, along with one research institute, FHU Institute for Comprehensive Medical Science (ICMS). In October 2020, FHU has celebrated its 52 years anniversary. Today, FHU consists of three schools, i. e., FHU School of Medicine, FHU School of Medical Sciences, and FHU School of Health Sciences, FHU Hospital, and one research institute, ICMS. The ICMS is a unique research institute with about 30 staffs dedicated to research. Dr. Fujita invited Prof. Dr. Yasuyuki Takagi, a pioneer of molecular biology in Japan, from Kyushu University to be the Director of ICMS in 1987. I succeeded Prof. Dr. Takagi as the Director of ICMS in April 1995, just a few weeks before the sudden death of Dr. Fujita in June 1995.

I herein present an overview of the scientific achievement of Dr. Fujita to commemorate his extraordinary contributions to medical science and education and share his legacy with all Fujita Health University as well as the general public, based on my memories from my research collaborations with him over 30 years.

## 3. The philosophy of President Dr. Fajita as the founding President of Fujita Gakuen and Fujita Health University in research and education

Dr. Fujita respected science as the basis of academia. “Our creativity for the people (*dokuso-ichiri*)”, is the School motto of Fujita Health University set by Dr. Fujita. He also emphasized the motto of FHU Hospitals in clinical practice: “We will treat suffering patients with INFINIT COMPASSION always with great humanity and modesty.” In his research activities, he also emphasized importance of cooperation with a modest attitude even during times of severe competition. In Table 1, I summarize the stated philosophy of Dr. Fujita in research, based on my experience from collaborations with him.

Dr. Fujita respected the research themes that were proposed based on the personal interests of each researcher, and he never forced researchers to work on some special projects. In my own experience when I was working at other universities (such as the Aichi Gakuin University School of Dentistry, the Tokyo Institute of Technology, and the Nagoya University School of Medicine), Dr.Fujita’s group and my group always collaborated on the themes in the mutually beneficial manner. He himself was interested in the biochemistry of diseases, that is, translational research to elucidate the molecular mechanism of diseases and to find the effective therapies to prevent and cure them. He also tried hard to educate and to develop research-minded medical scientists, physicians, and health science workers.

He proposed the necessity and the benefit of “interscience education and/or forums” for medical and health science workers, in order to promote close collaborations between them by avoiding hierarchies and sharing common information. He hoped to promote interscience collaborations with other universities and research institutes, not only in Japan but also abroad.

His philosophy that research is of great importance for the development of universities agrees well with the statement from August 2018 *Times Higher Education* (*THE*), which is a weekly magazine published in London, UK, that reported specifically on news and issues related to higher education: “If you have really good research-active people, they become the magnet for good students, good teaching, good academic reputation—all these things come together.”

In research, Dr. Fujita always used the most up-to-date methods and equipment: automatic protein analyzers, high-performance liquid chromatography (HPLC) with an electrochemical/fluorescence detector or mass spectrometer, and various types of the latest equipment for molecular biology/molecular genetics. He constructed SPF (specific-pathogen free) animal facilities for producing genetically engineered mice. He also introduced internet to his research facility in the early 1990s. Yoshikazu Kurosawa, Ph.D., who worked with Prof. Susumu Tonegawa (a Nobel laureate in 1987 behind the genetic principle for generation of antibody diversity) at the Massachusetts Institute of Technology (MIT), and Dr. Koichi Titani, who had worked as the Full Professor of Protein Chemistry at the University of Washington in Seattle in the 1970s, were invited as Professors to the FHU Institute for Comprehensive Medical Science (ICMS) in 1985. They contributed greatly to the introduction of the most advanced technology and research systems in molecular biology and protein chemistry.

**Figure 3:**
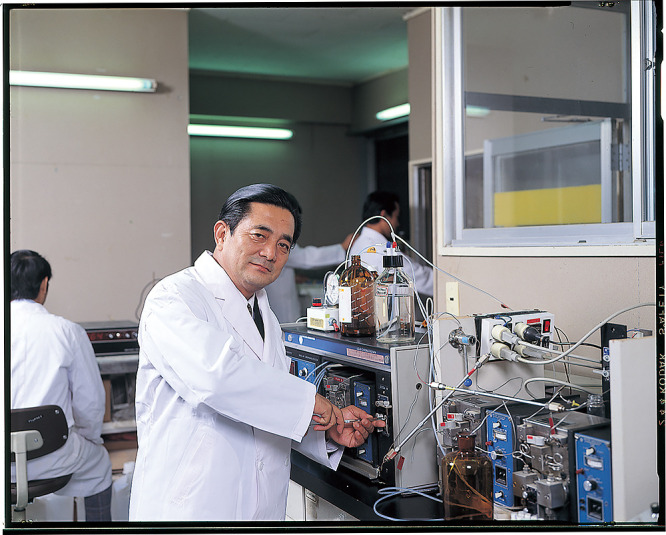
Dr. Keisuke Fujita working with high-performance liquid chromatography (HPLC).

Dr. Fujita also conducted critical experiments by himself, especially in his work on aloe plants at “Syoyaku Kenkyu Juku” (Center for Pharmacognosy) (Figure 3).

**Figure 4:**
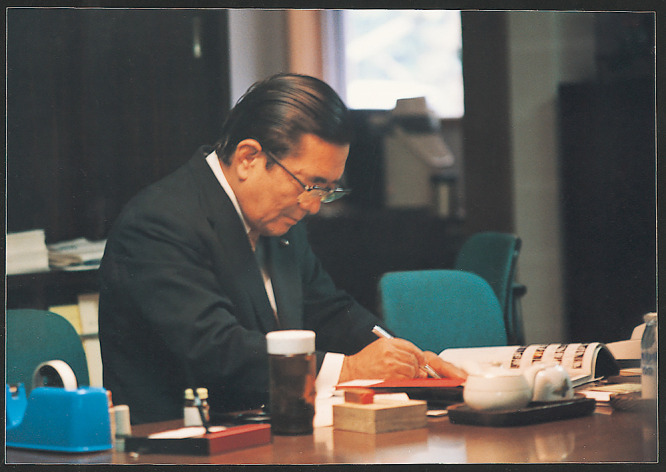
Dr. Keisuke Fujita writing a manuscript.

Dr. Fujita had excellent writing skills for scientific manuscripts. He often wrote papers late at night, even instead of his sleeping (Figure 4).

**Figure 5:**
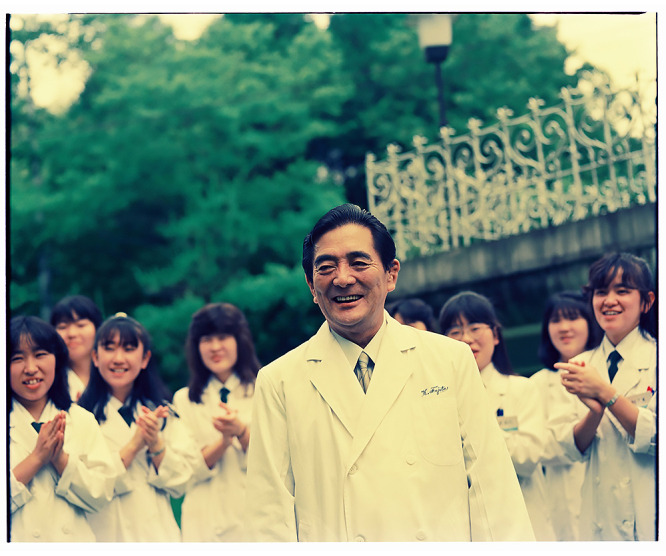
Dr. Keisuke Fujita after giving a lecture to students at FHU College.

**Figure 6:**
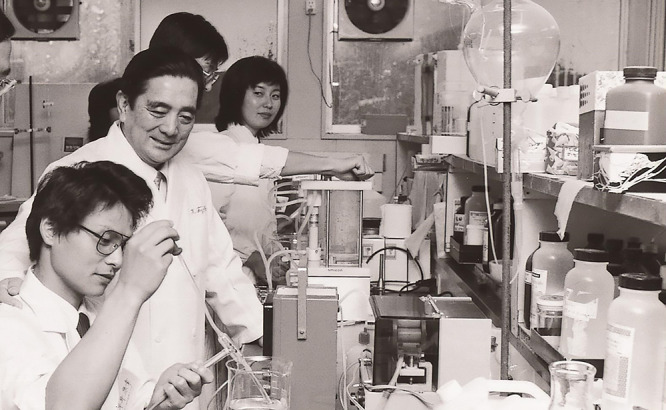
Dr. Keisuke Fujita in guiding an experiment in clinical chemistry.

Dr. Fujita was also an excellent mentor. During the early period of development at FHU, he presented lectures and guided experiments for students by himself (Figure 5; Figure 6). His attitude toward education was very clear, and he was a firm as a leader with great charisma, but he was always kind and warmhearted to his students. He respected the individuality of each student and always mentioned to the teaching staffs that “education” means to “educe” (bring out or develop) the capability of each student. By combining the efforts of all of the teaching staff at FHU through his strong leadership, Dr. Fujita created original and unique textbooks for various fields in medicine, health sciences, and medical biochemistry, containing many unique schemes and figures for students at the FHU School of Hygiene (now the FHU School of Medical Sciences and the FHU School of Health Sciences) and the FHU School of Medicine. This unique education system developed by Dr. Fujita has made a major contribution to the high success rate of FHU graduates in various national board examinations in the healthcare fields in Japan.

## 4. Selected publications of Dr. Keisuke Fujita

Dr. Fujita published 154 papers in international journals with high impact factors and 24 reviews published in various international conference proceedings, over the 40 years period from 1955 to 1995. These papers have been cited 5233 times and in 2020, his papers had an h-index of 35, which indicates that 35 manuscripts were cited over 35 times. The high number of citations and the h-index of 35 indicate the high impact of publications. It is rare for a founder and top leader of a university responsible for active administrator duties like Dr. Fujita to publish so many scientific papers in international journals and proceedings with high impact factors.

One hundred and eleven selected papers published in international journals with high impact factors from among his 154 papers are shown in Table 2 as a reference. The names of many collaborators are shown as co-authors.

### 4.1. Cancer

In the 1950s, while Dr. Fujita was the Assistant Professor of Biochemistry at the Nagoya University School of Medicine, he published three papers on cancer in “*Nature*.” Most of his papers on cancer were published in “*Cancer Research*”, the official journal of the American Association for Cancer Research, and “*Clinical Chemistry*” from the American Association for Clinical Chemistry. The selected papers on cancer are presented in Table 2 (1–4, 10, 13, 19, 23, 77, 111).

### 4.2. Clinical chemistry

Twelve selected papers on the clinical/analytical chemistry of various diseases, such as cancer, immune disorders including rheumatoid arthritis and systemic lupus erythematosus, and other diseases, such as stress-related disorders and hypertension, are presented in Table 2 (11, 15, 33, 40, 41, 43–45, 48, 55, 56, 66). These selected papers were published mainly in “*Clinical Chemistry*” from the American Association for Clinical Chemistry and “*Journal of Chromatography*” and “*Clinica Chimica Acta*” from Elsevier B.V.

### 4.3. Polyamines as biomarkers of cancer

Six selected papers on polyamines in blood and urine as early biomarkers of cancers are presented in Table 2 (6, 13, 17, 29, 57, 67). Although polyamines are found to be non-specific biomarkers in cancer, their increased levels in blood and urine are detected in early stages of various cancers. These selected papers were published in “*Cancer Research*” and “*Clinical Chemistry*.”

### 4.4. Pharmacognosy of Aloe plants

Dr. Fujita was extremely interested in aloe plants as an herbal medicine. Table 2 includes selected papers on aloe as an herbal medicine (5, 12, 16, 91, 92–96, 105, 106, 110). Aloe leaves (Aloe *arborescens* Miller var. *natalensis* Berger) have been used as an herbal medicine and a food supplement in Japan since the Edo period 200 years ago. Dr. Fujita grew aloe plants by himself, and prepared tablets for a dietary supplement from the freeze-dried homogenate of fresh aloe leaves. He obtained patents in Japan and in the USA on a method of preparing freeze-dried tablets of aloe as a dietary supplement. Aloin had been identified as a chemical component of aloe that is effective for constipation, but most of the other components of aloe with various therapeutic effects were unknown. He thus investigated the pharmacologically active ingredients of aloe. He identified carboxypeptidase as an enzyme effective for inflammation. We have received numerous requests to reprint papers on aloe as a dietary supplement, even as recently as in September 2020. Other important work performed by Dr. Fujita was the isolation and identification of the aloe lectin, which is considered to have anti-cancer activity. His papers on aloe plants were published in “*Phytotherapy Research*,” “*Biochemical Pharmacology*,” “*Biochemical Biophysical Research Communications*,” “*Journal of Biochemistry*,” and “*Japanese Journal of Cancer Research*.”

### 4.5. Melanin and melanoma

Dr. Fujita was also interested in melanoma. Twenty-nine of his selected papers on melanin and melanoma are shown in Table 2 (18–21, 24, 28, 30–32, 34–37, 42, 46, 48–52, 58–61, 68, 74, 75, 98, 99). Dr. Shosuke Ito, now Professor Emeritus at the FHU School of Medical Sciences, is an international leader of melanin chemistry, and joined to Dr. Fujita’s Laboratory in 1977 to promote the research on melanin chemistry and melanoma. Dr. Ito greatly contributed to the work on melanin chemistry and melanoma. Together, they elucidated the biosynthetic pathway of melanin from tyrosine catalyzed by tyrosinase and the chemical structure of melanin. They also discovered the anti-melanoma effects of cysteinyldopa, 4-S-cysteaminylphenol and its analogues. Dr. Kazumasa Wakamatsu, who succeeded Dr. Ito in his position as Professor and now Professor Emeritus at the FHU School of Medical Sciences, is continuing the research on melanin with Prof. Ito, including recently published papers on melanin in “*Science Signaling*” and “*Science*” (Zhou et al., *Sci Signal* 2018; 11: eaau7987; Barret et al., *Science* 2019; 363: 499–501).”

Dopamine neurons in the substantia nigra and noradrenaline neurons in the locus coeruleus in the brain contain neuromelanin. After the passing of Dr. Fujita in 1995, we discovered that neuromelanin is synthesized by tyrosine hydroxylase (TH/ tyrosine 3-monooxygenase; Nagatsu T, Levitt M, Udenfriend S. *J Biol Chem* 1964; 239: 2910–2917) from dopamine, (Ikemoto K, Nagatsu I, Ito S, King RA, Nishimura A, Nagatsu T. Does tyrosinase exist in neuromelanin-pigmented neurons in the human substantia nigra? *Neurosci Lett* 1998; 253: 198–200). Syntheses of neuromelanin from dopamine in the substantia nigra dopamine neurons and from noradrenaline in the locus coeruleus noradrenaline neurons were confirmed by chemical analyses of neuromelanin by Dr. Wakamatsu and Dr. Ito (Wakamatsu et al., *J Neurochem* 2003; 86:1015–1023; Wakamatsu et al., *J Neurochem* 2015; 135:768–776).

### 4.6. Dopamine β-hydroxylase

Together with Dr. Fujita, we were interested in dopamine β-hydroxylase (DBH: dopamine β-monooxygenase) in body fluids (blood and cerebrospinal fluid (CSF)) as a biomarker of disorders of peripheral and central noradrenergic systems. Fourteen selected papers on DBH are shown in Table 2 (7–9, 14, 22, 25, 26, 38, 62, 76, 89, 100, 103). DBH synthesizes noradrenaline from dopamine in noradrenergic neurons. We discovered that DBH is released in the periphery from noradrenergic sympathetic neurons into blood, and into cerebrospinal fluid from brain noradrenergic neurons. We established a method for assaying DBH activity in human blood (Nagatsu T, Udenfriend S., *Clin Chem* 1972; 18: 930–933; Nagatsu T., *Clin Chem* 2009; 55: 193–194), and further cloned the human *DBH* gene (69). Humans have the highest DBH activity/protein in blood among mammals. During 1970s, 1980s and 1990s, we found that the blood DBH activity/protein levels in humans are highly variable but constant in healthy individuals (1–100 IU μmol/min/L; mean values ~40 IU); and a genetically determined trait. We found that low levels of DBH activity/protein in blood and cerebrospinal fluid could be a biomarker of neuropsychiatric diseases such as DBH deficiency, Parkinson’s disease, and psychosis. We also identified that mutation in the human *DBH* gene causes low enzyme activity. Dr. Fujita extensively examined DBH levels in blood and cerebrospinal fluid in neuropsychiatric patients. These papers were mainly published in “*Journal of Neurochemistry*”, the official journal of the International Society for Neurochemistry (ISN).

### 4.7. Catecholamines and tetrahydrobiopterin: molecular biology

Twenty-six of Dr. Fujita’s selected papers on the molecular biology of the enzyme systems synthesizing catecholamines (dopamine, noradrenaline, and adrenaline) from tyrosine including tetrahydrobiopterin (BH4), which is an essential cofactor of tyrosine hydroxylase, are shown in Table 2 (27, 39, 47, 53, 54, 63–65, 69–73, 78–90, 97, 100–104, 107–109). During the 1980s and the 1990s, my colleagues and I worked with Dr. Fujita on the molecular biology of all human enzymes catalyzing the biosynthesis of catecholamines from tyrosine. Catecholamines are important neurotransmitters and hormones in physiology and pathology including neuropsychiatric diseases such as Parkinson’s disease and psychosis, and in stress-related disorders such as hypertension. Dr. Kurosawa and Dr. Fujita greatly assisted my colleagues and me in these works in the molecular biology of catecholamines. We purified all of the four human enzymes catalyzing the biosynthesis of catecholamines biosynthesis, tyrosine hydroxylase (TH), aromatic L-amino acid decarboxylase (AADC; DOPA decarboxylase: DDC), dopamine β-hydroxylase (DBH), and noradrenaline (phenylethanolamine) N-methyltransferase (PNMT); produced their antibodies; and cloned all human genes encoding these four enzymes. We cloned four isoforms of human tyrosine hydroxylase (types 1–4; TH1–TH4) produced by alternative mRNA splicing from a single gene, and found that the human *TH* gene contains human-specific exon 2, which produces types 3 and 4. We also found that non-human primates (monkeys; e.g., chimpanzees and gorilla) have two isoforms (types 1–2), while all non-primate mammals, such as mice, the human type 1 enzyme. These studies were performed under intense competition with Dr. Jacques Mallet’s group in France. Dr. Mallet reported on multiple forms of human tyrosine hydroxylase in “*Nature*” in 1987. In the same year, we also published the same findings in “*Biochemical Biophysical Research Communications*,” and in “*Nucleic Acids Research*” and “*Journal of Biochemistry*,” in 1987 and 1988, respectively. Dr. Kazuto Kobayashi, now Professor at Fukushima Medical University, played major roles in these works. I believe that these studies could not have been performed without the collaboration of Dr. Kurosawa and Dr. Fujita. We also created transgenic mice and knockout mice of these enzymes to investigate the *in vivo* functions of catecholamines. These works were published in “*Neuron*” (81), “*Journal of Biological Chemistry*” (64, 103, 108, 109), “*Biochemistry*” (78, 88), and “*Proceedings of the National Academy of Sciences USA* (PNAS)” (85, 107).

We also cloned the human gene encoding GTP cyclohydrolase I (GCH1), first synthesizing enzyme of tetrahydrobiopterin (BH4) from GTP, the essential cofactor of tyrosine hydroxylase, and reported this work in “*Journal of Biological Chemistry*” in 1995 (109). We found that mutations in the human *GCH 1* gene cause autosomal dominant DOPA-responsive dystonia, Segawa’s disease, which had been clinically identified by Dr. Masaya Segawa in Tokyo in 1972. Dr Hiroshi Ichinose, now Professor at the Tokyo Institute of Technology, did major work on the discovery of *GCH1* as the causative gene of Segawa’s disease, and this was published in “*Nature Genetics*” in 1994 (102). These manuscripts were our last collaborative works with Dr. Fujita. He sent me a letter of congratulations on the acceptance of these papers just before his sudden death on June 11, 1995.

## 5. Conclusion

**Figure 7:**
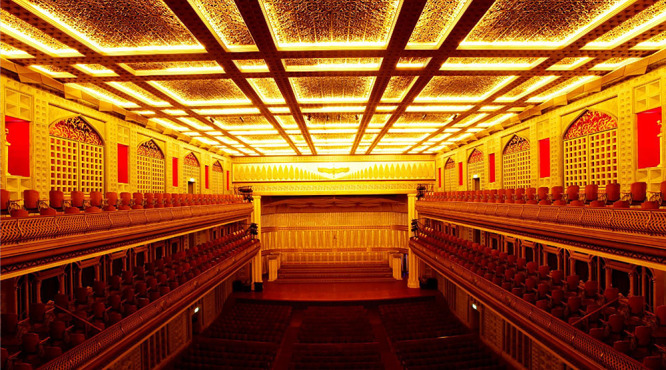
Fujita Hall 2000: interior.

**Figure 8:**
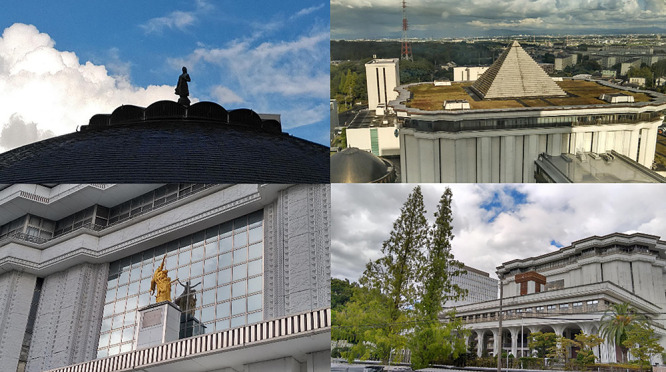
Fujita Hall 2000: exterior.

This article provides an overview of the life, work, and influence of Keisuke Fujita, M.D., Ph.D., the founding President of Fujita Gakuen, Fujita Health University (FHU), FHU School of Medicine, and FHU Institute for Comprehensive Medical Science (ICMS). Dr. Fujita made the extraordinary contributions to medical and health science education via the magnificent projects of establishing these three schools and one medical research institute of FHU. He was ambitious in creating a world-class university having the highest levels of education and research, in order to raise academically successful students with admirable personalities and great ingenuity. In: “Times Higher Education (THE): World University Rankings 2020”, FHU ranked number 8 among all universities and number 2 in private universities in Japan owing to the high ranks in citations/ publications. Even in death Dr. Fujita remains a major figure who contributed enormously to medicine, education and human welfare. Dr. Fujita was truly a man of science, yet he also had wide extracurricular interests in architecture, literature, music, the arts, and religion. In 1989, Dr. Fujita attended the ceremony of the 25th anniversary of Fujita Gakuen held in the newly constructed auditorium “Fujita Hall 2000,” the design of which was planned and constructed in collaboration with many specialists of architectures based on a conceptual design of Dr. Fujita himself (Figure 7, Figure 8).

Dr. Fujita, as a biochemist, thought that the hexagonal structure of benzene is a symbol of stability, and pursued the adoption of the hexagonal structures in several FHU buildings and many other places on campus. Dr. Fujita also promoted international and interdisciplinary collaborations. Many eminent scientists visited FHU: Dr. Sidney Udenfriend, the Roche Institute of Molecular Biology, Nutley, the USA (a member of the National Academy of Sciences of the USA); Dr. Julius Axelrod, NIH, Bethesda, the USA (Nobel laureate, 1970); Dr. Arvid Carlsson, Göteborg, Sweden (Nobel laureate, 2000); Dr. Marshall Nirenberg, NIH, Bethesda, USA (Nobel laureate, 1968); Dr. Leo Esaki, Tokyo, Japan (Nobel laureate, 1973); and Dr. Tasuku Honjo, Kyoto, Japan (Nobel laureate, 2018).

**Figure 9:**
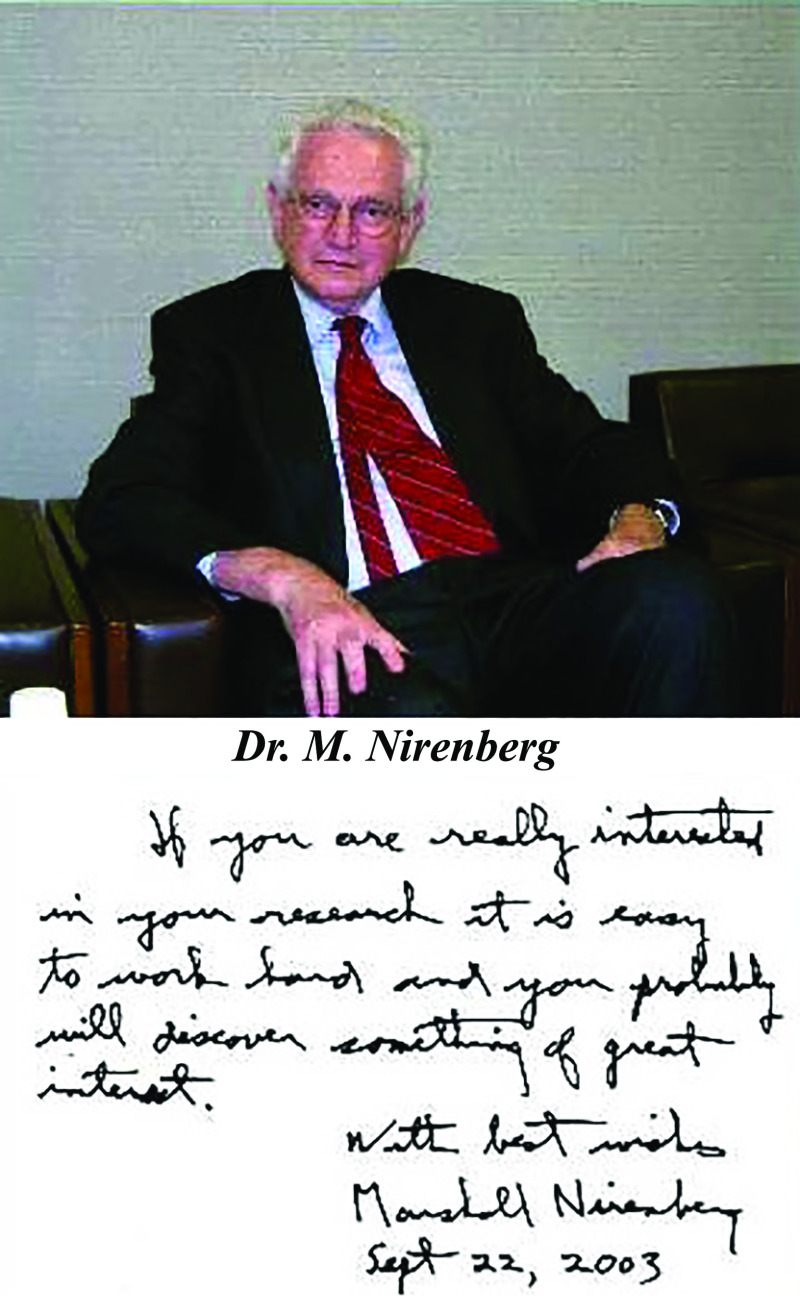
Dr. Marshall Nirenberg with his inspiring message to researchers at Fujita Health University in 2003.

Dr. Nirenberg left an inspiring message to researchers at FHU in 2003 (Figure 9): “If you are really interested in your research, it is easy for you to work hard and you probably will discover something of great interest.”

**Figure 10:**
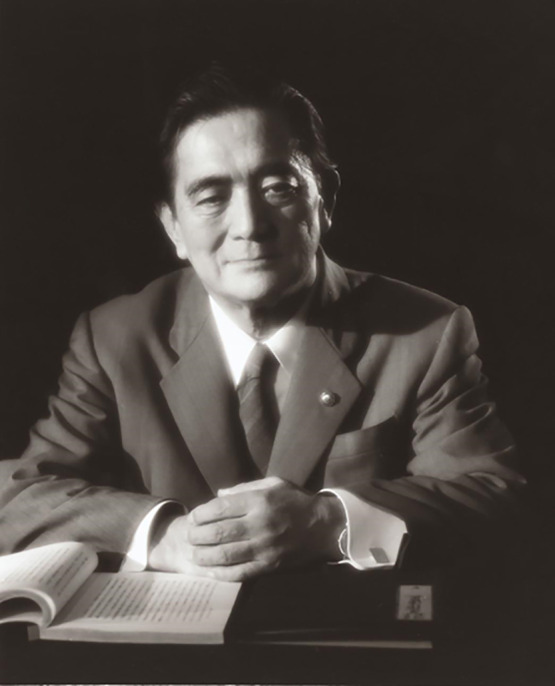
Dr. Keisuke Fujita in his late years.

It is assumed that someone has already made significant contributions to medical science. I hope that his future vision of FHU producing a Nobel Prize laureate will be realized (Figure 10).

## Figures and Tables

**Table1:** Proposed philosophy of Dr. Keisuke Fujita in research

•	Freedom in the selection of the research themes depending on the researcher’s own interests.
•	Promotion of research-minded medical and health science researchers (physician scientists).
•	Close collaboration between medical and health science researchers (“assembly”).
•	Translational research: Collaboration between basic scientists and physicians.
•	Collaboration with other domestic and international researchers.

**Table2:** Main publications by Dr. Keisuke Fujita

1) Iwase S, **Fujita K**.
Inhibiting effect of trypan blue on the experimental production of liver cancer.** Nature 1955**; 175: 552.
2) **Fujita K**, Mine T, Iwase S, MizunoT, Takayanagi T, Sugiyama Y, Arai T.
The carcinogenicity of certain compounds related to trypan blue. **Br J Exper Pathol 1957**; 38: 291–6.
3) **Fujita K**, Iwase S, Ito T, Matsuyama M.
Inhibiting effect of chlorpromazine on the experimental production of liver cancer. **Nature 1958**; 181: 54.
4) **Fujita K**, Mine T, Ito T, Matsuyama M.
Effects of certain compounds related to trypan blue on the experimental production of liver cancer. **Nature 1958**; 181: 1732–3.
5) **Fujita K**, Teradaira R, Nagatsu T.
Bradykininase activity of aloe extract. **Biochem Pharmacol 1976**; 25: 205.
6) **Fujita K**, Nagatsu T, Maruta K, Ito M, Senba H, Miki K.
Urinary putrescine, spermidine, and spermine in human blood and solid cancers and in an experimental gastric tumor of rats. **Cancer Res** **1976**; 36: 1320–4.
7) **Fujita K**, Nagatsu T, Maruta K, Teradaira R, Beppu H, Tsuji Y, Kato T.
Fluorescence assays for dopamine β-hydroxylase activity in human serum by high-performance liquid chromatography.** Anal Biochem** **1977**; 82: 130–40.
8) **Fujita K**, Maruta K, Teradaira R, Beppu H, Ikegame M, Nagatsu T, Kato T.
Normal values for serum dopamine β-hydroxylase activity. **Clin Chem 1977**; 23: 1947.
9) **Fujita K**, Maruta K, Teradaira R, Beppu H, Shinpo K, Maeno Y, Ito T, Nagatsu T, Kato, T.
Dopamine β-hydroxylase activity in human cerebrospinal fluid and serum.** J Neurochem 1977**; 29: 1141–2.
10) **Fujita K**, Yamada K, Sato T, Takagi I, Sakashita T.
A rapid method for embedding biological tissues in a low viscosity epoxy resin “Quetol 651” for electron microscopy. **J Electron Microsc 1977**; 26: 165–6.
11) **Fujita K**, Hirano M, Tokunaga K, Nagatsu I, Nagatsu T, Sakakibara, S.
Serum glycylproline *p*-nitroanilidase activity in blood cancers. **Clin Chim Acta 1977**; 81: 215–7.
12) **Fujita K**, Yamada Y, Azuma K, Hirosawa S.
Effect of leaf extracts of *Aloe arborescens* Mill, subsp. *natalensis* Berger on growth of Trichophyton mentagrophytes. **Antimicrob Agents Chemother 1978**; 14: 132–6.
13) **Fujita K**, Nagatsu T, Shinpo K, Maruta K, Takahashi H, Sekiya A.
Increase of urinary putrescine in 3,4-benzopyrene carcinogenesis and its inhibition by putrescine. **Cancer Res 1978**; 38: 3509–11.
14) **Fujita K**, Ito T, Maruta K, Teradaira R, Beppu H, Nakagami Y, Kato Y, Nagatsu, T, Kato T.
Serum dopamine β-hydroxylase in schizophrenic patients. **J Neurochem 1978**; 30; 1569–72.
15) **Fujita K**, Hirano M, Ochiai J, Funabashi M, Nagatsu I, Nagatsu T, Sakakibara S.
Serum glycylproline *p*-nitroanilidase activity in rheumatoid arthritis and systemic lupus erythematosus.** Clin Chim Acta 1978**; 88: 15–20.
16) **Fujita K**, Ito S, Teradaira R, Beppu H.
Properties of a carboxypeptidase from aloe.** Biochem Pharmacol 1979**; 28: 1261–2.
17) **Fujita K**, Nagatsu T, Shinpo K, Maruta K, Teradaira R, Nakamura M.
Improved analysis for urinary polyamines by use of high voltage electrophoresis on paper. **Clin Chem 1980**; 26: 1577–82.
18) Ito S, Teradaira R, **Fujita K**.
Distribution and metabolism of tritium-labelled 5-*S*-cysteinyldopa in mice. **Biochem Pharmacol 1980**; 29: 3277–80.
19) **Fujita K**, Ito S, Inoue S, Yamamoto Y, Takeuchi J, Shamoto M, Nagatsu T.
Selective toxicity of 5-*S*-cysteinyldopa, a melanin precursor, to tumor cells *in vitro* and *in vivo*. **Cancer Res 1980**; 40: 2543–6.
20) Ito S, **Fujita K**.
Formation of cysteine conjugates from dihydroxyphenylalanine and its *S*-cysteinyl derivatives by peroxidase-catalyzed oxidation. **Biochim Biophys Acta 1981**; 672: 151–7.
21) Ito S, Inoue S, Yamamoto Y, **Fujita K**.
Synthesis and antitumor activity of cysteinyl-3,4-dihydroxyphenylalanines and related compounds. **J Med Chem 1981**; 24: 673–7.
22) Matsui H, Kato T, Yamamoto C, **Fujita K**, Nagatsu T.
Highly sensitive assay for dopamine β-hydroxylase activity in human cerebrospinal fluid by high performance liquid chromatography-electrochemical detection: Properties of the enzyme. **J Neurochem 1981**; 37: 289–96.
23) **Fujita K**, Shinpo K, Yamada K, Sato T, Niimi H, Shamoto M, Nagatsu T, Takeuchi T, Umezawa H.
Reduction of adriamycin toxicity by ascorbate in mice and guinea pigs. **Cancer Res 1982**; 42: 309–16.
24) Ito S, **Fujita K**.
Conjugation of dopa and 5-*S*-cysteinyldopa with cysteine mediated by superoxide radical. **Biochem Pharmacol 1982**; 31: 2887–9.
25) **Fujita K**, Maruta K, Teradaira R, Beppu H, Ikegame M, Kawai K, Nagatsu T.
Dopamine β-hydroxylase activity in human cerebrospinal fluid from various age groups.** Clin Chem 1982**; 28: 1403–4.
26) **Fujita K**, Teradaira R, Inoue T, Takahashi H, Beppu H, Kawai K, Maruta K, Yagyu S, Nagatsu T.
Stress-induced changes in *in vivo* and *in vitro* dopamine β-hydroxylase activity in spontaneously hypertensive rats. **Biochem Med 1982**; 28: 340–6.
27) Oka K, Sekiya M, Osada H, **Fujita K**, Kato T, Nagatsu T.
Simultaneous fluorometry of urinary dopamine, norepinephrine, and epinephrine compared with liquid chromatography with electrochemical detection. **Clin Chem 1982**; 28: 646–9.
28) Ito S, Maruta K, Imai Y, Kato T, Ito M, Nakajima S, **Fujita K**, Kurahashi T.
Urinary *p*-aminobenzoic acid determined in the pancreatic function test by liquid chromatography with electrochemical detection. **Clin Chem 1982**; 28: 323–6.
29) Shinpo K, **Fujita K**, Maruta K, Teradaira R, Nagatsu T.
Comparative measurements of urinary polyamines in early morning and 24-hour urine specimens. **Clin Chim Acta 1983**; 131: 143–8.
30) Ito S, Inoue S, **Fujita K**.
The mechanism of toxicity of 5-*S*-cysteinyldopa to tumour cells. Hydrogen peroxide as a mediator of cytotoxicity. **Biochem Pharmacol 1983**; 32: 2079–81.
31) Ito S, Homma K, Kiyota M, **Fujita K**, Jimbow K.
Characterization of structural properties for morphologic differentiation of melanosomes. Ⅲ. Free and protein-bound dopa and 5-*S*-cysteinyldopa in B16 and Harding-Passey melanomas.** J Invest Dermatol 1983**; 80: 207–9.
32) Ito S, Jimbow K, Kato T, Kiyota M, **Fujita K**.
Protein-bound dopa and 5-*S*-cysteinyldopa in non-melanogenic tissues.** Acta Derm Venereol (Stockh) 1983**; 63: 463–7.
33) **Fujita K**, Maruta K, Ito S, Nagatsu T.
Urinary 4-hydroxy-3-methoxymandelic (vanillylmandelic) acid, 4-hydroxy-3-methoxyphenylacetic (homovanillic) acid, and 5-hydroxy-3-indoleacetic acid determined by liquid chromatography with electrochemical detection.** Clin Chem 1983**; 29: 876–7.
34) Ito S, **Fujita K**.
Oxygen-dependent conjugation of dopa with cysteine catalyzed by iron-EDTA complex.** Biochem Pharmacol 1984**; 33: 2193–7.
35) Ito S, **Fujita K**, Takahashi H, Jimbow K.
Characterization of melanogenesis in mouse and guinea pig hair by chemical analysis of melanins and of free and bound dopa and 5-*S*-cysteinyldopa. **J Invest Dermatol 1984**; 83: 12–4.
36) Ito S, Kato T, Maruta K, **Fujita K**.
Determination of DOPA, dopamine, and 5-*S*-cysteinyl-DOPA in plasma, urine, and tissue samples by high-performance liquid chromatography with electrochemical detection. **J Chromatogr 1984**; 311: 154–9.
37) Ito S, Kato T, Shinpo K, **Fujita K**.
Oxidation of tyrosine residues in proteins by tyrosinase. Formation of Protein-bonded 3,4-dihydroxyphenylalanine and 5-*S*-cysteinyl-3,4-dihydroxyphenylalanine. **Biochem J 1984**; 222: 407–11.
38) Matsui H, Kato T, Yamamoto C, **Fujita K**, Sakai H, Nagatsu T.
A sensitive fluorometric assay for dopamine β-hydroxylase activity by high-performance liquid chromatography. **Biochem Med 1984**; 31: 140–6.
39) Ikeda M, Hirata Y, **Fujita K**, Shinzato M, Takahashi H, Yagyu S, Nagatsu T.
Effects of stress on release of dopamine and serotonin in the striatum of spontaneously hypertensive rats: An *In vivo* voltammetric study.** Neurochem Int 1984**; 6: 509–12.
40) Nagatsu T, Ikeda M, Hirata Y, **Fujita K**, Takahashi H, Shinzato M, Yagyu S.
Abnormality in catecholamine metabolism in the brain of spontaneously hypertensive rats (SHR)-analysis by *In vivo* voltammetry.** Jpn Heart J 1984**; 25: 820–2.
41) Maruta K, **Fujita K**, Ito S, Nagatsu T.
Liquid chromatography of plasma catecholamines, with electrochemical detection, after treatment with boric acid gel. **Clin Chem 1984**; 30: 1271–3.
42) Ito S,** Fujita K**.
Microanalysis of eumelanin and pheomelanin in hair and melanomas by chemical degradation and liquid chromatography. **Anal Biochem 1985**; 144: 527–36.
43) Nagatsu I, Ito M, Kawakami Y, Karasawa N, Takahashi H, **Fujita K**, Nagatsu T.
Quantitative immunofluorescence of tyrosine hydroxylase in the adrenal medulla of spontaneously hypertensive rats. **Experientia 1985**; 41: 1054–5.
44) Iwase-Okada K, Nagatsu T, **Fujita K**, Torikai K, Hamamoto T, Shibata T, Maeno Y, Sakakibara S.
Serum collagenase-like peptidase activity in rheumatoid arthritis and systemic lupus erythematosus. **Clin Chim Acta 1985**; 146: 75–9.
45) Ito S, Kato T, Maruta K, Jimbow K, **Fujita K**.
“Total” acidic metabolites of catecholamine in urine as determined by hydrolysis with hydriodic acid and liquid chromatography: application to patients with neuroblastoma and melanoma. **Clin Chem 1985**; 31: 1185–8.
46) Kato T, Ito S, **Fujita K**.
Tyrosinase-catalyzed binding of 3,4-dihydroxyphenylalanine with proteins through the sulfhydryl group. **Biochim Biophys Acta 1986**, 881, 415–21.
47) Togari A, Murakami T, Oshima T, **Fujita K**, Nagatsu, T.
Effects of polyamines on tyrosine hydroxylase activity in adrenals. **Neurochem Int 1986**; 9: 281–6.
48) Ito S, **Fujita K**, Yoshioka M, Sienko D, Nagatsu T.
Identification of 5-*S* and 2-*S*-cysteinyldopamine and 5-*S*-glutathionyldopamine formed from dopamine by high-performance liquid chromatography with electrochemical detection. **J Chromatogr 1986**; 375: 134–40.
49) Ito S, Kato T, **Fujita K**.
Determination of urinary 5-*S*-cysteinyldopamine by high-performance liquid chromatography with electrochemical detection. **Chromatographia 1986**; 21: 645–7.
50) Inoue S, Ito S, Imai Y, Kasuga T, **Fujita K**.
Growth inhibition of melanoma cells by *N*-protected dopa derivatives. **Biochem Pharmacol 1987**; 36: 3537–40.
51) Ito S, Kato T, **Fujita K**.
Seasonal variation in urinary excretion of 5-*S*-cysteinyldopa in healthy Japanese. **Acta Derm Venereol (Stockh) 1987**; 67: 163–5.
52) Miura S, Ueda T, Jimbow K, Ito S, **Fujita K**.
Synthesis of cysteinylphenol, cysteaminylphenol, and related compounds, and *in vivo* evaluation of antimelanoma effect. **Arch Dermatol Res 1987**; 279: 219–25.
53) Kaneda N, Kobayashi K, Ichinose H, Kishi F, Nakazawa A, Kurosawa Y, **Fujita K**, Nagatsu T.
Isolation of a novel cDNA clone for human tyrosine hydroxylase alternative RNA splicing produces four kinds of mRNA from a single gene. **Biochem Biophys Res Commun** **1987**; 146: 971–5.
54) Kobayashi K, Kaneda N, Ichinose H, Kishi F, Nakazawa A, Kurosawa Y, **Fujita K**, Nagatsu, T.
Isolation of a full length cDNA clone encoding human tyrosine hydroxylase type 3. **Nucleic Acids Res 1987**; 15: 6733.
55) Kojima K, Mihara R, Sakai T, Togari A, Matsui T, Shinpo K, **Fujita K**, Fukasawa K, Harada M, Nagatsu T.
Serum activities of dipeptidyl-aminopeptidase II and dipeptidyl-aminopeptidase IV in tumor-bearing animals and in cancer patients. **Biochem Med Metab Biol** **1987**; 37: 35–41.
56) Imai Y, Ito S, **Fujita K**.
Determination of natural thiols by liquid chromatography after derivatization with 3,5-di-tert-butyl-1,2-benzoquinone. **J Chromatogr 1987**; 420: 404–10.
57) Watanabe N, Asano M, Yamamoto K, Nagatsu T, Matsumoto T, **Fujita K**.
Electrochemical detection of polyamines using immobilized enzyme as postcolumn reactor in high performance liquid chromatography. **Chemistry Lett 1988**: 1169–70.
58) Naoi M., Takahashi T, Ito S, **Fujita K**, Nagatsu T.
Accumulation of *N*-methyl-4-phenylpyridinium ion (MPP^+^) in human melanoma cell line, HMV- I and - II. **Neurosci Lett 1988**; 87: 57–62.
59) Ito S, Imai Y, Jimbow K, **Fujita K**.
Incorporation of sulfhydryl compounds into melanins *in vitro*. **Biochim Biophys Acta 1988**; 964: 1–7.
60) Wakamatsu K, Ito S, **Fujita K**.
Melanin-related metabolites in urine of B16 melanoma-bearing mice.** Acta Derm Venereol (Stockh) 1988**; 68: 385–9.
61) Ito S, Kato T, **Fujita K**.
Covalent binding of catechols to proteins through the sulphydryl group. **Biochem Pharmacol 1988**; 37: 1707–10.
62) Mogi M, Harada M, Kojima K, Inagaki H, Kondo T, Narabayashi H, Arai T, Teradaira R, **Fujita K**, Kiuchi K, Nagatsu T.
Sandwich enzyme immunoassay of dopamine β-hydroxylase in cerebrospinal fluid from control and parkinsonian patients.** Neurochem Int 1988**; 12: 187–91.
63) Kobayashi K, Kaneda N, Ichinose H, Kishi F, Nakazawa A, Kurosawa Y, **Fujita K**, Nagatsu T.
Structure of the human tyrosine hydroxylase gene: Alternative splicing from a single gene accounts for generation of four mRNA types. **J Biochem 1988**; 103: 907–12.
64) Kaneda N, Ichinose H, Kobayashi K, Oka K, Kishi F, Nakazawa A, Kurosawa Y, **Fujita K**, Nagatsu T.
Molecular cloning of cDNA and chromosomal assignment of the gene for human phenylethanolamine *N*-methyltransferase, the enzyme for epinephrine biosynthesis. **J Biol Chem 1988**; 263: 7672–7.
65) Kobayashi K, Kiuchi K, Ishii A, Kaneda N, Kurosawa Y, **Fujita K**, Nagatsu, T.
Expression of four types of human tyrosine hydroxylase in COS cells. **FEBS Lett 1988**; 238: 431–4.
66) Imai Y, Ito S, Maruta K, **Fujita K**.
Simultaneous determination of catecholamines and serotonin by liquid chromatography, after treatment with boric acid gel. **Clin Chem 1988**; 34: 528–30.
67) Maruta K, Teradaira R, Watanabe N, Nagatsu T, Asano M, Yamamoto K, Matsumoto T, Shionoya Y, **Fujita K**.
Simple, sensitive assay of polyamines by high-performance liquid chromatography with electrochemical detection after post-column reaction with immobilized polyamine oxidase. **Clin Chem 1989**; 35: 1694–6.
68) Ito S, Wakamatsu K, Inoue S, **Fujita K**.
Correlation between urinary melanin-related metabolites and tumour weight in melanoma-bearing mice. **Acta Derm Venereol (Stockh) 1989**; 69: 380–4.
69) Kobayashi K, Kurosawa Y, **Fujita K**, Nagatsu T.
Human dopamine β-hydroxylase gene: two mRNA types having different 3'-terminal regions are produced through alternative polyadenylation. **Nucleic Acid Res 1989**; 17: 1089–102.
70) Uchida K, Takamatsu K, Kaneda N, Toya S, Tsukada Y, Kurosawa Y, **Fujita K**, Nagatsu T, Kohsaka S.
Synthesis of L-3,4-dihydroxyphenylalanine by tyrosine hydroxylase cDNA-transfected C6 cells: application for intracerebral grafting. **J Neurochem 1989**; 53: 728–32.
71) Ichinose H, Kurosawa Y, Titani K, **Fujita K**, Nagatsu T.
Isolation and characterization of a cDNA clone encoding human aromatic L-amino acid decarboxylase. **Biochem Biophys Res Commun 1989**; 164: 1024–30.
72) Sasaoka T, Kaneda N, Kurosawa Y, **Fujita K**, Nagatsu T.
Structure of human phenylethanolamine *N*-methyltransferase gene: existence of two types of mRNA with different transcription initiation sites. **Neurochem Int 1989**; 15: 555–65.
73) Mogi M, Harada M, Kojima K, Adachi T, Narabayashi H, **Fujita K**, Naoi M, Nagatsu T.
β2-Microglobulin decrease in cerebrospinal fluid form parkinsonian patients. **Neurosci Lett 1989**; 104: 241–6.
74) Inoue S, Ito S, Wakamatsu K, Jimbow K, **Fujita K**.
Mechanism of growth inhibition of melanoma cells by 4-*S*-cysteaminylphenol and its analogues. **Biochem Pharmacol 1990**; 39: 1077–83.
75) Wakamatsu K, Ito S, **Fujita K**.
Production, circulation, and excretion of melanin-related metabolites in B16 melanoma-bearing mice.** Acta Derm Venereol (Stockh) 1990**; 70, 367–72.
76) Nagatsu I, Kobayashi K, Fujii T, Komori K, Sekiguchi K, Titani K, **Fujita K**, Nagatsu T.
Antibodies raised against different oligopeptide segments of human dopamine β-hydroxylase. **Neurosci Lett 1990**; 120: 141–5.
77) Shinpo K, Nagatsu T, Yamada K, Sato T, Niimi H, Shamoto M, Takeuchi T, Umezawa H, **Fujita K**.
Ascorbic acid and adriamycin toxicity.** Am J Clin Neutr 1991**; 54: 1298S–1301S.
78) Kiuchi K, Kiuchi K, Titani K, **Fujita K**, Suzuki K, Nagatsu T.
Limited proteolysis of tyrosine hydroxylase by Ca^2+^-activated neutral protease (Calpain). **Biochemistry 1991**; 30: 10416–9.
79) Ichinose H, Katoh S, Sueoka T, Titani K,** Fujita K**, Nagatsu T.
Cloning and sequencing of cDNA encoding human sepiapterin reductase—An enzyme involved in tetrahydrobiopterin biosynthesis—. **Biochem Biophys Res Commun 1991**; 179: 183–9.
80) Yamada K, Sakai M, Takeuchi T, Karasawa N, Kaneda N, Sasaoka T, Kobayashi K, Yokoyama M, Nomura T, Katsuki M,** Fujita K**, Nagatsu T, Nagatsu I.
Enhanced expression of human tyrosine hydroxylase in the lower brainstem of transgenic mice. **Neurosci Lett 1991**; 134: 57–61.
81) Kaneda N, Sasaoka T, Kobayashi K, Kiuchi K, Nagatsu I, Kurosawa Y, **Fujita K**, Yokoyama M, Nomura T, Katsuki M, Nagatsu T.
Tissue-specific and high-level expression of the human tyrosine hydroxylase gene in transgenic mice.** Neuron 1991**; 6: 583–94.
82) Nagatsu I, Yamada K, Karasawa N, Sasaoka T, Kobayashi K, Yokoyama M, Nomura T, Katsuki M, **Fujita K**, Nagatsu T.
Expression in brain sensory neurons of the transgene in transgenic mice carrying human tyrosine hydroxylase gene. **Neurosci Lett 1991**; 127: 91–5.
83) Ogiwara S, Nagatsu T, Teradaira R, **Fujita K**, Sugimoto T.
Occurrence of umanopterin, a new diastereomer of neopterin, in urine from cancer patients. **Tetrahedron Lett 1992**; 33: 1341–2.
84) Ichinose H, Katoh S, Sueoka T, Titani K,** Fujita K**, Nagatsu T.
Cloning and sequencing of cDNA encoding human sepiapterin reductase. **Pteridines** **1992**; 3: 41–2.
85) Kobayashi K, Sasaoka T, Morita S, Nagatsu I, Iguchi A, Kurosawa Y, **Fujita K**, Nomura T, Kimura M, Katsuki M, Nagatsu T.
Genetic alteration of catecholamine specificity in transgenic mice. **Proc Natl Acad Sci USA 1992**; 89: 1631–5.
86) Togari A, Ichinose H, Matsumoto S, **Fujita K**, Nagatsu T.
Multiple mRNA forms of human GTP cyclohydrolase I. **Biochem Biophys Res Commun 1992**; 187: 359–65.
87) Ogiwara S, Kiuchi K, Nagatsu T, Teradaira R, Nagatsu I, **Fujita K**, Sugimoto T.
A highly sensitive and specific enzyme liked immunoadsorbent assay of neopterin and biopterin in biological samples. **Clin Chem 1992**; 38: 1954–8.
88) Ichinose H, Sumi-Ichinose C, Ohye T, Hagino Y, **Fujita K**, Nagatsu T.
Tissue-specific alternative splicing of the first exon generates two types of mRNAs in human aromatic L-amino acid decarboxylase. **Biochemistry 1992**; 31: 11546–50.
89) Nakano T, Kobayashi K, Saito S, **Fujita K**, Nagatsu T.
Mouse dopamine β-hydroxylase-primary structure deduced from the cDNA sequence and exon/intron organization of the gene. **Biochem Biophys Res Commun 1992**; 189: 590–9.
90) Nagatsu T, Ichinose H, Katoh S, **Fujita K**, Titani K.
6R-tetrahydrobiopterin biosynthesis: structures of rat and human sepiapterin reductase. **J Nutr Sci Vitaminol Special Issue 1992**: 510–3.
91) Kawai K, Beppu H, Koike T, **Fujita K**, Marunouchi T.
Tissue culture of *Aloe arborescens* Miller var. *natalensis* Berger. **Phytother Res 1993**; 7: S5–10.
92) Ito S, Teradaira R, Beppu H, Obata M, **Fujita K**, Nagatsu T.
Biochemical properties of carboxypeptidase from *Aloe arborescens* Miller var. *natalensis* Berger. **Phytother Res 1993**; 7: S26–9.
93) Obata M, Ito S, Beppu H, **Fujita K**, Nagatsu T.
Mechanism of antiinflammatory and antithermal burn action of CPase from *Aloe arborescens* Miller var. *natalensis* Berger in rats and mice.** Phytother Res 1993**; 7: S30–3.
94) Teradaira R, Shinzato M, Beppu H, **Fujita K**.
Antigastric ulcer effects in rats of *Aloe arborescens* Miller var. *natalensis* Berger extract. **Phytother Res 1993**; 7: S34–6.
95) Beppu H, Nagamura Y,** Fujita K**.
Hypoglycaemia and antidiabetic effects in mice of *Aloe arborescens* Miller var. *natalensis* Berger. **Phytother Res 1993**; 7: S37–42.
96) Tsuda H, Matsumoto K, Ito M, Hirano I, Kawai K, Beppu H,** Fujita K**, Nagao M.
Inhibitory effect of *Aloe arboresens* Miller var. *natalensis* Berger (Kidachi aloe) on induction of preneoplastic focal lesions in the rat liver.** Phytother Res 1993**; 7: S43–7.
97) Ogiwara S, Hidaka H, Sugimoto T, Teradaira R, **Fujita K**, Nagatsu T.
Elevated levels of oncopterin, *N^2^*-(3-aminopropyl) biopterin, a new pterin compound, in urine from patients with solid and blood cancers.** J Biochem 1993**; 113: 1–3.
98) Hasegawa K, Inoue S, Wakamatsu K, Ito S, **Fujita K**, Ishikawa K.
Changes in plasma 5-*S*-cysteinyldopa concentration in B16 melanoma-bearing mice treated with interferon-β or dacarbazine. **Melanoma Res 1993**; 3: 377–80.
99) Inoue S, Hasegawa K, Ito S, Wakamatsu K, **Fujita K**.
Antimelanoma activity of chloroquine, an antimalarial agent with high affinity for melanin. **Pigment Cell Res1993**; 6: 354–8.
100) Morita S, Kobayashi K, Mizuguchi T, Yamada K, Nagatsu I, Titani K, **Fujita K**, Hidaka H, Nagatsu T.
The 5'-flanking region of the human dopamine β-hydroxylase gene promotes neuron subtype-specific gene expression in the central nervous system of transgenic mice. **Mol Brain Res 1993**; 17: 239–44.
101) Nomura T, Ichinose H, Sumi-Ichinose C, Nomura H, Hagino Y, **Fujtia K**, Nagatsu T.
Cloning and sequencing of cDNA encoding mouse GTP cyclohydrolase I. **Biochem Biophys Res Commun 1993**; 191: 523–7.
102) Ichinose H, Ohye T, Takahashi E, Seki N, Hori T, Segawa M, Nomura Y, Endo K, Tanaka H, Tsuji S, **Fujita K**, Nagatsu T.
Hereditary progressive dystonia with marked diurnal fluctuation caused by mutations of the GTP cyclohydrolase I gene. **Nature Genetics 1994**; 8: 236–42.
103) Kobayashi K, Morita S, Mizuguchi T, Sawada H, Yamada K, Nagatsu I, **Fujita K**, Nagatsu T.
Functional and high-level expression of human dopamine β-hydroxylase in transgenic mice. **J Biol Chem** 1993; 269: 29725–31.
104) Mogi M, Harada M, Riederer P, Narabayashi H, **Fujita K**, Nagatsu T.
Tumor necrosis factor-α (TNF-α) increases both in the brain and in the cerebrospinal fluid from parkinsonian patients. **Neurosci Lett** 1994; 165: 208–10.
105) Koike T, Beppu H, Kuzuya H, Maruta K, Shimpo K, Suzuki M, Titani K, **Fujita K**.
A 35 kDa mannose-binding lectin with hemagglutinating and mitogenic activities from “Kidachi Aloe” (*Aloe arborescens* Miller var. *natalensis* Berger). **J Biochem 1995**; 118: 1205–10.
106) Koike T, Titani K, Suzuki M, Beppu H, Kuzuya H, Maruta K, Shimpo K, **Fujita K**.
The complete amino acid sequence of a mannose-binding lectin from “Kidachi Aloe” (*Aloe arborescens* Miller var. *natalensis* Berger). **Biochemical and Biophysical Research Communications 1995**; 214: 163–70.
107) Kobayashi K, Morita S, Sawada H, Mizuguchi T, Yamada K, Nagatsu I,** Fujita K**, Kreitman RJ, Pastan I, Nagatsu T.
Immunotoxin-mediated conditional distraction of specific neurons in transgenic mice. **Proc Natl Acad Sci USA 1995**; 92: 1132–6.
108) Kobayashi K, Morita S, Sawada H, Mizuguchi T, Yamada K, Nagatsu I, Hata T, Watanabe Y, **Fujita K**, Nagatsu T.
Targeted distribution of the tyrosine hydroxylase locus results in severe catecholamine depletion and peripheral lethality in mice. **J Biol Chem 1995**; 270: 27235–43.
109) Ichinose I, Ohye T, Matsuda Y, Hori T, Blau N, Burlina A, Rouse B, Matalon R, **Fujita K**, Nagatsu T.
Characterization of mouse and human GTP cyclohydrolase I gene. Mutations in rat and patients with GTP cyclohydrolase I deficiency. **J Biol Chem 1995**; 270: 10062–71.
110) Uehara N, Iwahori Y, Asamoto M, Baba-Toriyama H, Iigo M, Ochiai M, Nagao M, Nakayama M, Degawa M, Matsumoto K, Hirono I, Beppu H, **Fujita K**, Tsuda H.
Decreased levels of 2-amino-3-methylimidazo[4,5-*f*]quinoline-DNA adducts in rats treated with β-carotene, α-tocopherol and freeze-dried *Aloe*. **Japan J Cancer Res 1996**; 87: 342–8.
111) Shimpo K, Takahashi H, Tsuda H, Hibino T, Kawai K, Kimura C, Nagatsu T, **Fujita K**.
Inhibition of hepatocellular carcinoma development and erythrocyte polyamine levels in ODS rats fed on 3'-methyl-4-dimethylaminoazobenzene by hemicalcium ascorbate, 2-O-octadecylascorbic acid, and ascorbyl palmitate.** Cancer Detect Prev 1996**; 20: 137–45.

